# Maize intercropping in the milpa system. Diversity, extent and importance for nutritional security in the Western Highlands of Guatemala

**DOI:** 10.1038/s41598-021-82784-2

**Published:** 2021-02-12

**Authors:** Santiago Lopez-Ridaura, Luis Barba-Escoto, Cristian A. Reyna-Ramirez, Carlos Sum, Natalia Palacios-Rojas, Bruno Gerard

**Affiliations:** 1 International Maize and Wheat Improvement Center (CIMMYT), El Batan, Texcoco, Mexico; 2 Universidad Autónoma Metropolitana-Xochimilco (UAM-X), Doctorado en Ciencias Biológicas y de la Salud, Ciudad de México, Mexico; 3 BuenaMilpa Project, International Maize and Wheat Improvement Center (CIMMYT), Quetzaltenango, Guatemala

**Keywords:** Evolution, Ecology, Environmental sciences, Health care

## Abstract

We present an assessment of the extent, diversity, and nutritional contribution of the milpa through a quantitative analysis of data from a survey conducted in 989 small scale farm households in the Western Highlands of Guatemala (WHG). The milpa is a traditional agricultural system in which maize is intercropped with other species, such as common beans, faba beans, squashes or potatoes. Our study shows that more than two-thirds of the 1,205 plots recorded were under the milpa system, with a great diversity of crop combinations. As shown with the 357 plots for which specific yields were available, milpa systems present higher total productivity than monocropped maize, expressed as total energy yield of the harvested crops in the respective system, and were also better at providing the recommended daily allowances of fourteen essential nutrients, based on a Potential Nutrient Adequacy (PNA) indicator. Maize-bean-potato, maize-potato, and maize-bean-faba intercrops had the highest PNAs, and monocropped maize, the lowest. These results support the implementation of milpa systems tailored to different agro-ecologies in order to improve nutrition in the WHG and a variety of similar regions.

## Introduction

**Figure 1:**
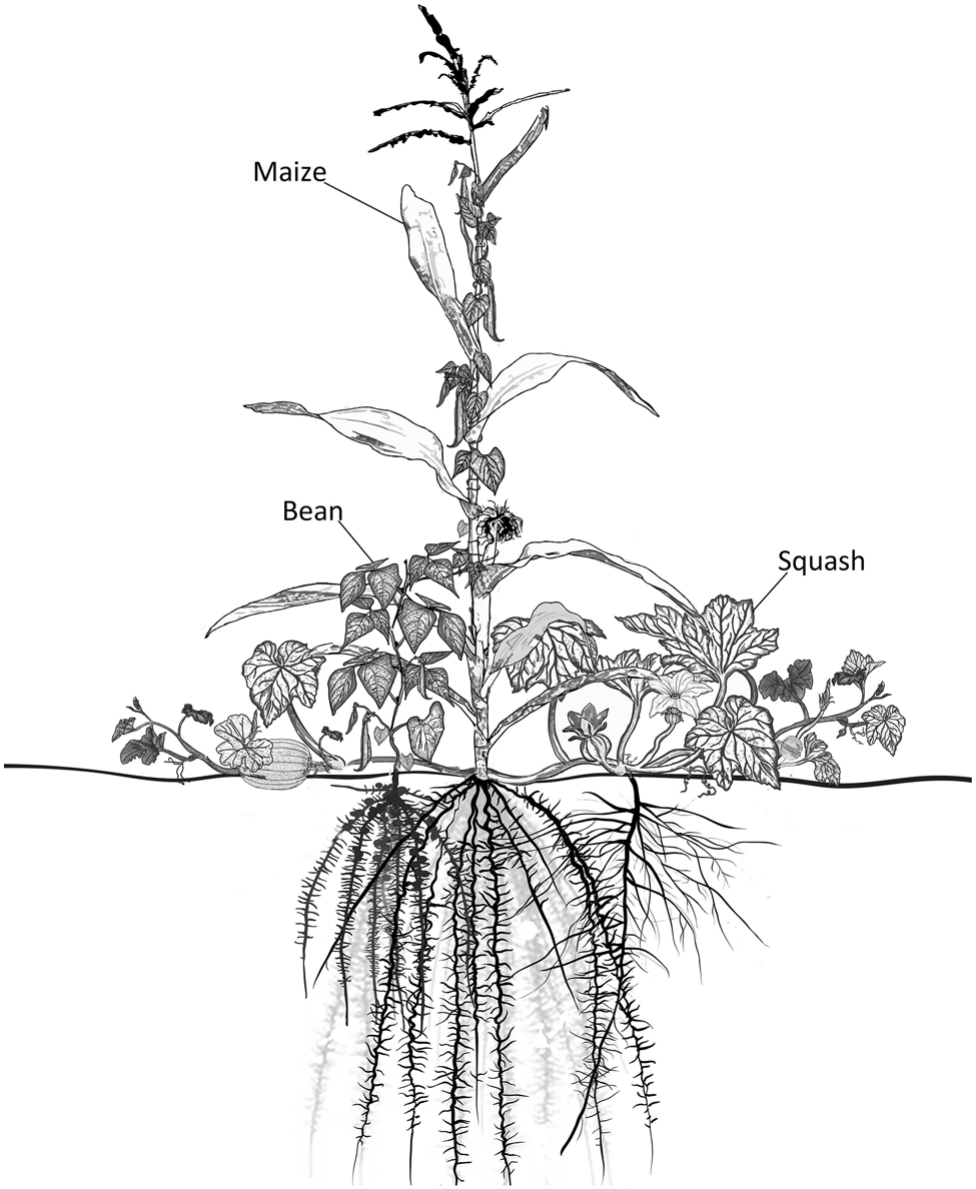
The classic milpa with maize-bean-squash.

Maize (*Zea mays*) is one of the most widely cultivated crops in the world, produced on almost 200 million hectares in practically all countries of the world^1^. In Mesoamerica, its center of origin and diversity^2,3^ farmers have traditionally intercropped maize with several other species in what is known as the “milpa” or “three sisters” system^4,5^. The milpa typically comprises maize intercropped with common beans (*Phaseolus* spp.) and squashes (*Cucurbita* spp.) (Fig. 1)^6^. Archaeological and historical evidence identifies the milpa as the backbone of agriculture in pre-Columbian times in the vast region spanning from northeast North America to southern Central America^4,5,7^.

Farmers throughout Mesoamerica continue to grow maize in variants of the milpa system in a range of agro-ecologies from arid and semiarid zones to the temperate highlands and tropical lowlands. In addition to being intercropped with common beans and squash, maize may be grown with faba beans (*Vicia faba*), peppers (*Capsicum* spp.), tomato (*Solanum lycopersicum*), potato (*Solanum tuberosum*) and amaranth (*Amaranthus* spp.), among others, and even with wild leafy species used as food or for medicinal purposes^8^. The degree of variation in the milpa systems depends on different factors including climate, soil type, topography, natural vegetation, traditional knowledge, culture and diets.

Studies of the total productivity of milpas grown under partially controlled conditions, have shown them to be an example of efficient traditional cropping system. In milpa systems, niche complementarity, competition, and facilitation among species, contribute synergistically to overall performance^7,9,10^, chiefly through the efficient use of land, water, nutrients, and light^11,12^. In the classic maize-bean-squash milpa, for example, the maize stalk supports the climbing bean, increasing the latter’s access to light, while the bean plant fixes additional nitrogen in the soil. The squash shades the soil surface, reducing moisture loss and impeding weed growth (Fig. 1).

The Western Highlands of Guatemala (WHG) is among the world’s poorest regions and food insecurity and malnutrition affect more than half of its inhabitants^13,14^. Recent research has highlighted the milpa’s critical role as a source of food and nutritional security^15,16^, providing both macro (starch, protein, fat) and micro-nutrients (vitamins and minerals). This contribution of the milpa to nutrition is especially relevant for small-scale farm households and communities such as the WHG, whose inhabitants consume nearly all they produce^13,17^.

Here we report on the extent and diversity of milpa systems in the WHG using data collected on a survey of 989 farm households located across 59 villages of the region. We also present a quantitative analysis to assess, at the plot level, the potential contribution of the different milpa systems to food and nutritional security, by determining the number of persons a given area of milpa can adequately feed. With data from all the 357 plots for which there were specific yield values, we calculated, at plot level, both the total crop productivity (expressed as total energy yield of the harvested crops in the respective system) and the Potential Nutrient Adequacy (PNA), which is a recently developed indicator based on ecological niche theory^18^. To our knowledge, this is the first report on the relation between the milpa intercropping diversity and its nutritional capacity, at least the first that takes on account data from more than one or a few plots or crop combinations. We believe the results of this analysis can inform a variety of research and development efforts oriented to improve the lives of rural families in the WHG and similar regions.

## Results

### Milpa extent and diversity

**Figure 2:**
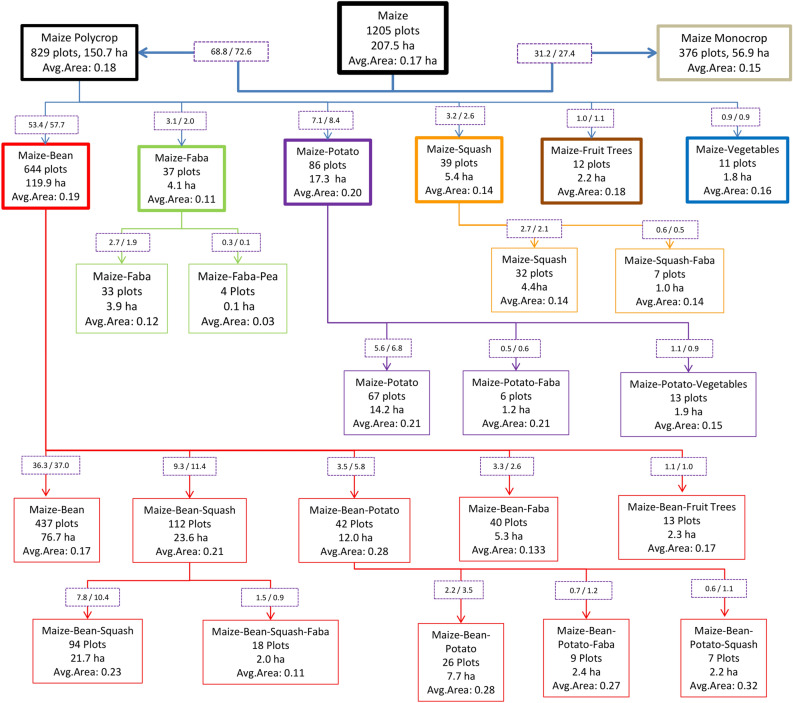
Maize plots reported in the survey of farm households in the Western Highlands of Guatemala. Milpa systems and their varied crop combinations are represented in different colours. The tree is nested, so the first node displays all maize plots, with successive splits for monocrop vs intercropped maize and with crop names presented according to how often they were grown (overall: maize > common beans > potatoes > squash > faba beans > fruit trees > vegetables). Numbers in the boxes superimposed on the arrows are the percentages, of number of plots and area under the respective milpa system, of the total 1 205 maize plots.

The 989 households surveyed, reported a total of 1,541 plots, with an overall combined area of 297.8 ha, where maize, coffee, potato, and other crops were grown. The average agricultural area per household is 0.30 ha (median 0.17 ha). The survey took in only farm households that grew maize in at least one of their plots (see section “Data”), but the importance of maize in local diets can be nonetheless appreciated in that 78% of the plots (1205) were used to grow it. Of the 1,205 maize plots, 829 of them (69% of the plots and 73% of the maize area) included maize in association (or milpas) with at least one other crop, among them common beans, faba beans, potato, squashes, fruit trees, coffee and/or vegetables. Maize in monocrop was found on 376 plots (31% of the plots and 27% of the maize area), and those plots were significantly smaller than the milpa plots, with average areas of 0.15 vs 0.18 ha (Fig. 2), respectively.

The 829 milpa plots showed diverse cropping combinations including maize with beans on 78% of the plots, with potato on 10%, and with faba bean on about 5%. In 24% of the milpa plots (203) maize was associated with two other crops while in 4% of the cases (34) it was associated with other three crops. The most common milpa included maize-beans (644 plots), 68% these plots featured only these two crops while the rest were further diversified with squash (17%), potato (6%) and faba bean (6%) (Fig. 2).

### Milpa yield performance and contribution to food security

**Figure 3:**
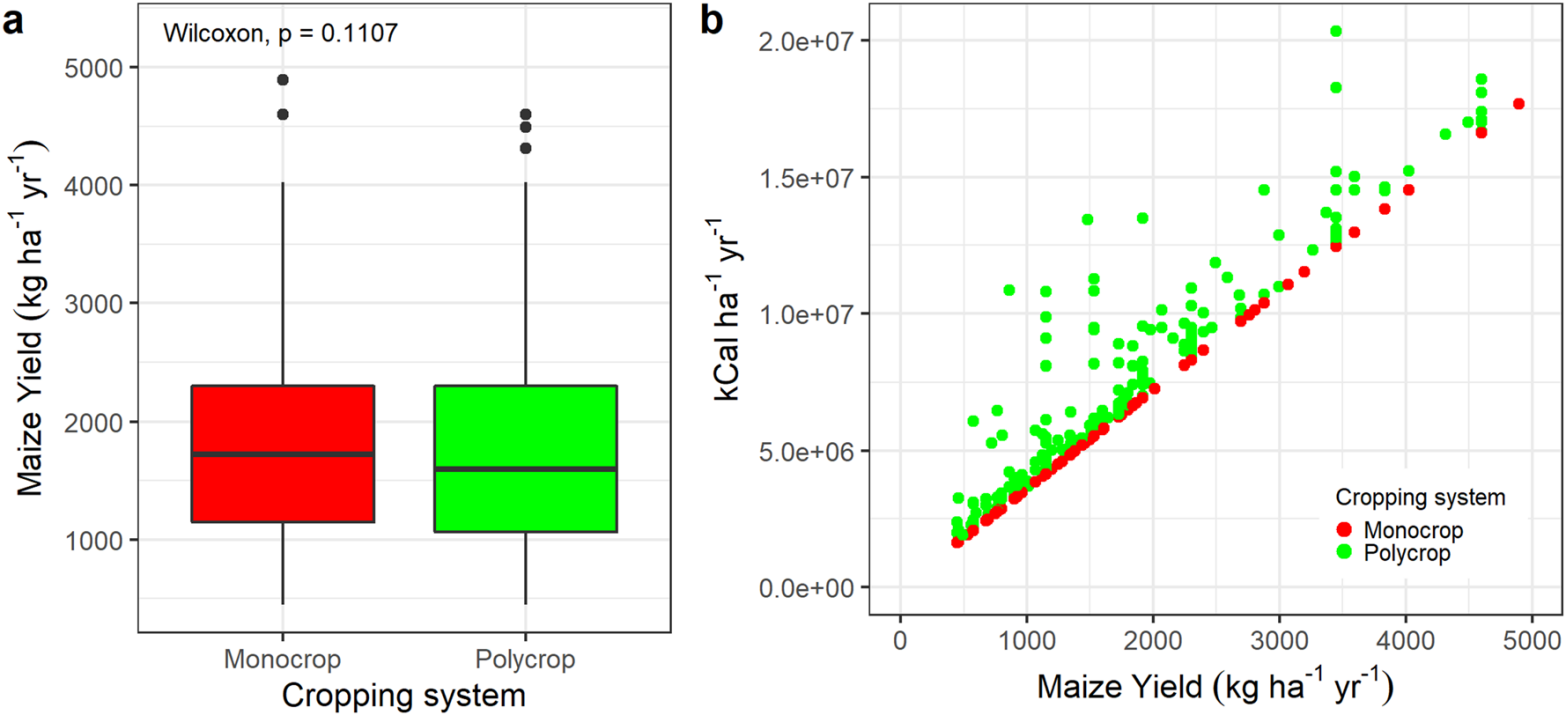
Maize yield (**a**) and total caloric yields vs maize yield (**b**) under maize monocrop and milpa systems (polycrop) in the Western Highlands of Guatemala.

Because of the structure of the survey applied we could only calculate yield values for the different crops reported from 357 plots (i.e. crop production in one section of the survey and area and cropping patterns of each plot in another section, therefore discarding for the analysis all farmers with more than one plot as it was not possible to calculate yield at the plot level- See section “Milpa and food security”). Maize grain yields were very variable in both maize monocrop and the milpa systems and were lower under the milpa system, averaging 1801 kg ha^−1^ (sd = 1011), compared to monocropped maize (average of 1981 kg ha^−1^, sd = 1099) but was not significantly different (Wilcoxon test, W = 17,390, p-value = 0.107) (Fig. 3a) suggesting no yield penalty when maize is associated with other crops found in the milpa systems. The total caloric yield (calculated based on the kCal content of different crops—See section “Potential adequacy of milpas for human nutrition” and Supplementary Tables 3 and 4) of milpa systems was consistently higher than that of the maize monocrop (Fig. 3b), providing more food energy per unit area than sole maize.

**Figure 4:**
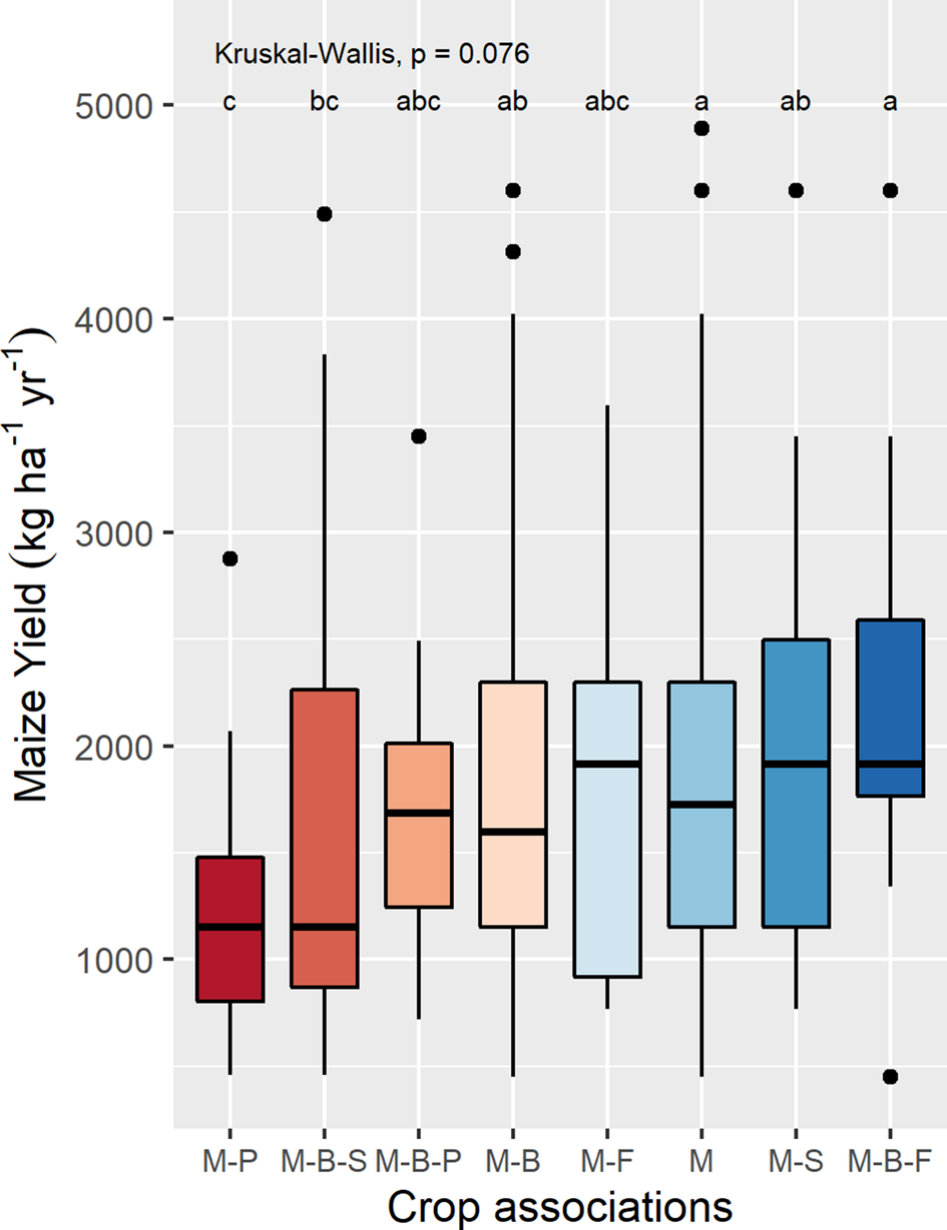
Maize yield of milpa systems in the Western Highlands of Guatemala. Crop combinations are ordered by their mean yield. Significantly different groups after Dunn’s test are indicated with letters within the graphs. Crop associations codes are: M-P: Maize-Potato, M-B-S: Maize-Bean-Squash, M-B-P: Maize-Bean-Potato, M-B: Maize-Bean, M-F: Maize-Faba, M: Maize monocrop, M-S: Maize-Squash, M-B-F: Maize-Bean-Faba.

Average maize grain yield for the different milpa systems ranged from 1233 kg ha^−1^ for maize-potato to 2320 kg ha^−1^ for maize-bean-faba. After pairwise *post-hoc* comparisons (Dunn’s test), some significant differences on maize yield can be found among crop combinations, being maize-potato and maize-bean-squash significantly lower than maize in monocrop and maize-bean-faba (Fig. 4, Supplementary Table 2). However, because of the difference in sample size for the different intercropping systems, the results should be taken with caution.

### Nutrition contribution and nutrient adequacy of the milpa system

**Figure 5:**
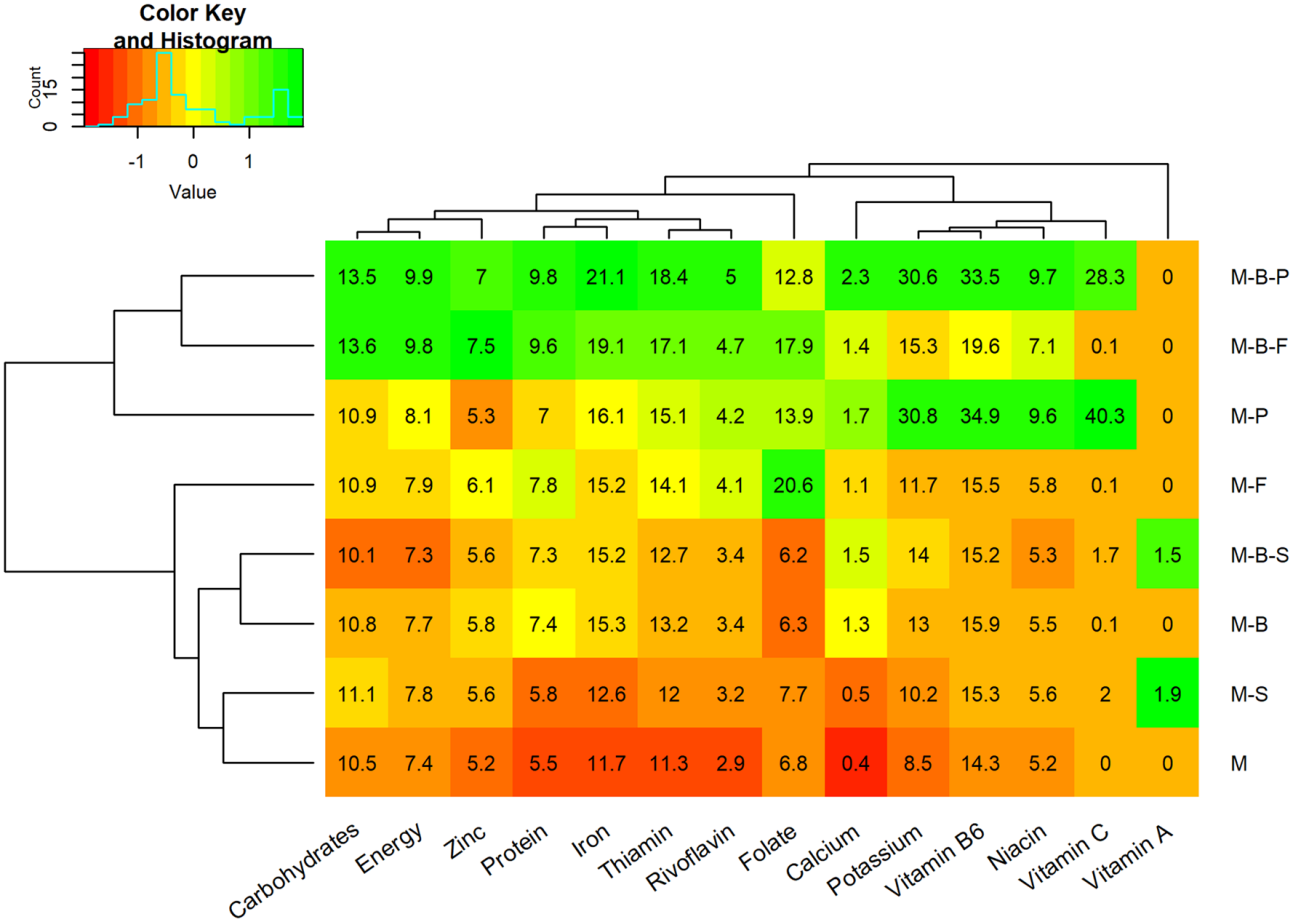
Heatmap showing the Potential Number of People Fed (PNPF) per ha per year (numbers in the cells) considering the recommended daily allowances for key human nutrients, for a range of milpa systems in the Western Highlands of Guatemala. Crop associations codes are: M-P: Maize-Potato, M-B-S: Maize-Bean-Squash, M-B-P: Maize-Bean-Potato, M-B: Maize-Bean, M-F: Maize-Faba, M: Maize monocrop, M-S: Maize-Squash, M-B-F: Maize-Bean-Faba. The colour-code denotes a gradient (of scaled PNPF values) from high PNPF in green to low PNPF in red, crop associations and nutrients are clustered in a dendrogram (in black to the left and top respectively) according to the PNPF values (Created using *heatmap.2* function from the *R* package *gplots* -http://cran.r-project.org/web/packages/gplots/index.html).

The nutritional functional diversity (NFD) of the milpa systems will always be higher than NFD of a maize monocrop, as other crops provide additional nutrients. NFD increases with crop species diversity and especially when the additional crops come from different botanical families. Assuming that milpa is the sole food provider, the potential number of people fed (PNPF) was calculated considering the essential components of human nutrition, the nutrient concentrations in the common edible parts of the raw crops, and the amounts of each crop produced. In this study, PNPF was highest for three intercrops: maize-bean-potatoes, maize-bean-faba and maize-potatoes (Fig. 5). The maize-bean-squash, maize-bean and maize-faba intercrops showed an intermediate PNPF for most nutrients and maize-squash and maize in monocrop had the lowest PNPF values for most nutrients.

**Figure 6:**
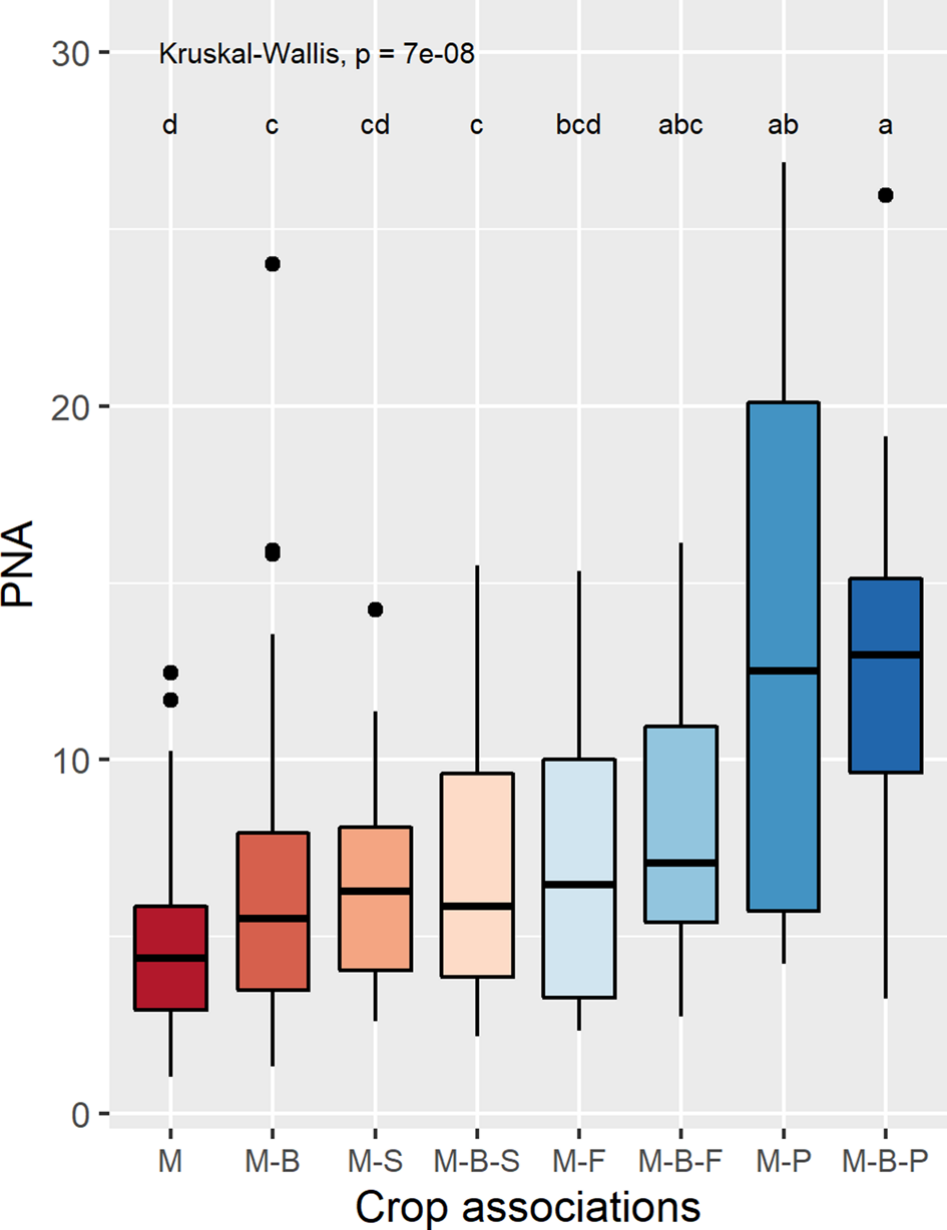
The Potential Nutrient Adequacy (PNA) of milpa systems in the Western Highlands of Guatemala. Crop combinations are ordered by their PNA mean value. Dunn’s test significant groups are indicated by letters within the graph. Crop associations codes are: M: Maize monocrop, M-B: Maize-Bean, M-S: Maize-Squash, M-B-S: Maize-Bean-Squash, M-F: Maize-Faba, M-B-F: Maize-Bean-Faba, M-P: Maize-Potato, M-B-P: Maize-Bean-Potato.

The Potential Nutrient Adequacy (PNA) represents the balanced level of nutrients provided by each crop combination considering all nutrients and their recommended daily allowances (See Sect. 4.4). The different milpa systems showed significant differences in their PNA values (Kruskal–Wallis Chi-sq. = 46.5, p < 7.01 e-08) (Fig. 6). After Dunn’s post hoc test, the PNA of the maize monocrop was found to be significantly below most of other PNA values while the PNA for maize-bean-faba, maize-potato, and maize-bean-potato were significantly higher than the rest. The PNA for maize-bean, maize-squash, maize-bean-squash and maize-faba were in the mid-range and did not show significant differences (Fig. 6).

## Discussion and conclusions

This study showed the extent and diversity of the milpa system and its potential contribution to the nutritional security of small-scale farm households in the WHG. It highlights the importance of milpa system in the WHG and the advantages of maize intercropping over mono-cropping in subsistence farming systems. It also highlights the nutritional potential of the different milpa combinations that are traditionally grown in WHG. Whereas the classic milpa features a maize-bean-squash intercrop, in the WHG maize-bean was the most common combination with variants that included faba bean, potatoes, peas, vegetables and fruit trees. This likely reflects the range of altitude (1,400 to 3,200 masl) and corresponding agroclimatic variation in the WHG, but especially farmers’ choices based on their needs, means, market opportunities, knowledge and traditions.

The milpa system is the classic example of an efficient multi/mixed-cropping system, which tends to be more productive and efficient in use of light, nutrients and water than monocrop systems, given its internal dynamics of complementarity, competition and facilitation. For example, the mechanisms of interspecific root interactions where maize root exudates promote nodulation of the faba bean, making maize-faba intercrops more efficient than their monocrops have been described^19^.

The Land Equivalent Ratio (LER) is a common way to measure the yield advantage of multi vs monocropping systems^20^. In field experiments mimicking the traditional Iroquoian three sisters system in the north-eastern USA, LERs of between 1.06 and 1.3 were found^7^. Under experimental conditions in Central Mexico, LERs for the maize-bean-squash milpa of between 1.6 and 1.9, which corresponds to 60% and 90% greater total productivity than a maize monocrop, were reported^10^. Even higher yield advantages have been reported for the milpa system when integrated with fruit trees^21^. LERs were not calculated in this study due to the lack of robust information on the performance of all crops (e.g. common beans, squash, faba bean, potatoes) grown in monoculture. However, given that our study found no significant yield difference for maize when grown alone or with other crops, we can consider that the LER for the milpa systems in the WHG would be, in most cases, greater than or equal to 1.0. Further analysis, possibly including more targeted data collection and experimentation, could help identifying best crop combinations in specific agro-ecologies for improved agronomic performance of milpa systems.

Our results show higher nutritional output of the milpa systems over monocropped maize in the WHG. This is based on the Potential Nutrient Adequacy (PNA), which takes into account not only the diversity of nutrients and nutrient sources, as does the dietary diversity index^22^, but also includes (1) how much is produced by each crop and their nutrient concentrations and (2) the recommended daily intake for each nutrient, thus providing a more complete estimation of the potential contribution of the milpa system to the nutrition security of farm households in the WHG. Dietary diversity studies, defined as the number of food groups consumed, have shown that increasing farming diversity often increases the nutrient adequacy of the human diet^23^. The PNA of the milpa system thus offers a good estimation of its contribution to nutritional security in the WHG and supports the idea that dietary diversity and crop and animal species richness in farm households are positively correlated^24^.

Beyond yield and calories, milpa systems produced significantly more other essential nutrients. The maize-bean-faba, maize-potatoes and maize-bean-potatoes associations had highest PNAs, contributing the most carbohydrates, proteins, zinc, iron, calcium, potassium, folate, thiamin, riboflavin, vitamin B6, niacin and vitamin C. Similar results showed that, in a trial implemented in USA, the maize-bean-squash intercrop provided more calories and proteins than a maize monocrop^15^. Based on a case study with one family farm in southern Mexico, it was shown that an average family with the average amount of land in a Mayan community could meet the daily nutritional requirements for fat, carbohydrates, fiber, protein, vitamins A and C, calcium, iron, zinc, and niacin, through diets based on products from the milpa^16^. One important limitation to highlight in these studies, as well as the one presented here, is the fact that all calculations are based on the production and concentration of nutrients in their raw form; the nutritional contribution of the cooked food considering consumption patterns (portions size and frequency), the nutritional contributions of food obtained outside the milpa system such as poultry, livestock and home-gardens or purchased food, and the storage of milpa products and associated effects on nutrient stability need to be studied^25,26^. Equally important is the variation in the availability of milpa products throughout the year, given crops’ seasonality.

This study focused on the plot level, i.e. characterizing the diversity of milpa systems and their production of different nutrients. Results are shown at the hectare and yearly basis to allow comparison and use of standard indicators for assessing the performance of cropping systems. For example, in Fig. 5 our results show that one hectare of the different crop combinations can provide enough protein to satisfy the needs of between 5.5 and 9.8 male adults for a year, while for iron these values range between 11.7 and 21.1 (i.e. the Potential Number of People Fed (PNPF)). However, to better grasp the contribution of the milpa systems to the nutritional security of subsistence farmers households in the WHG, it is needed to take into account that (1) most farm households in the WHG have less than a quarter of a hectare (an average of 0.30 and a median of 0.17 for the households surveyed) and that (2) the average number of people per household (comprising all ages) in the survey is six. Thus, despite the advantages of some milpa systems to provide more nutrients per unit of land, the generalised low land availability for small scale farmers in the WHG makes it impossible for the milpa system alone to satisfy the needs of all household members. This structural problem of land availability and general marginalization of indigenous communities in the WHG has been widely documented and is one of the main causes of the endemic poverty and malnutrition in the region^13,14,17^. Farmers in the WHG have been forced to find alternative food sources through, for example, the production of cash crops (such as non-traditional export vegetables) and buy staple crops with the money generated or/and national and international migration to generate enough earning to complement their own production of food.

In conclusion, our study shows the great extent and diversity of milpa systems in the WHG. Total food productivity and nutrient functional diversity advantages in milpa systems, over maize in monocrop, were found in small-scale farm households in the WHG. These findings highlight the importance of this traditional cropping system for smallholder farmers’ nutritional security in the region. The milpa system alone has not, and will not, completely satisfy food demand for farm households in the WHG but, together with other sources of food either produced on-farm or purchased, can contribute importantly to improve the nutritional situation of this impoverished and marginalised region. Depending on specific characteristics of the farm households, their agro-ecological conditions and their availability of different sources of food and nutrients, milpa systems can be tailored to improve the food and nutritional security of small-scale farmers in the WHG and in a wide range of similar regions.

## Methods

### Data

Data were collected in 2015 through a survey deployed as part of the Feed the Future Buena Milpa project (http://www.cimmyt.org/project-profile/buena-milpa/). The project’s goal was to reduce food insecurity and malnutrition by fostering sustainable, resilient, and innovative maize-based farming systems in the WHG.

We conducted the survey, with support from local researchers and agronomy students of the University of San Carlos (USAC), in summer of 2015 in 989 maize-growing farm households in 64 communities of 16 municipalities of the WHG. The number of surveys at the departmental level was: Totonicapán (226), Quiche (350), Quetzaltenango (187) and Huehuetenango (226) (See Supplementary Table 1 and Supplementary Fig. 1 for details). Criteria to select communities were: location within the targeted municipalities in the Buena Milpa project, the active work of project partners within the communities, and their distribution within four selected watersheds that included farming systems at different altitudes, ranging from 1400 to 3200 masl. For household selection, enumerators walked radial transects to survey household members, choosing only households that explicitly agreed to participate and had agricultural land on at least part of which maize was grown. The survey was a closed questionnaire with 139 questions in 18 sections including 5 project themes: milpa-maize germplasm improvement, natural resource conservation in farming system, farming system diversification, agricultural innovation systems and social inclusion.

### Milpa diversity and extent

We used survey findings regarding the previous year’s crop production (2014) for all 989 surveyed households to understand the importance and diversity of the milpa system. The 989 households reported crop production on a total of 1,541 plots, 1,324 of which included maize. The other 217 plots grew potato (85 plots), coffee (50), vegetables (31), bean (19), fruit trees (12), faba bean (7), forestry trees (6), pea (2), oats, wheat, etc. Given the study’s focus on smallholder farmers, we discarded other 119 plots that had unrealistically large values for plot size or maize production levels for the region (i.e. more than ten times the average plot size or calculated maize yield, > 2 ha and > 11 ton ha^−1^ year^−1^).

For the resulting 1205 plots we constructed a tree depicting maize-system diversity in the WHG, with each node indicating the main cropping associations. The tree was nested, so the first node displays all maize plots, with successive splits for monocrop vs intercropped maize and with crop names presented according to how often they are grown (overall: maize > common beans > potatoes > squash > faba beans > fruit trees > vegetables). So, in plots where maize is grown with potatoes and common beans, the association is termed *maize-bean-potatoes*.

### Milpa and food security

We assessed maize yield differences for monocropping and intercropping to detect a yield penalty or advantage for maize (see below), including survey information only for households from which we had available yield data for all crops. In the survey, total production for each crop was recorded regardless of the number of plots on which it was grown and therefore, for 398 households (40.2% of the sample) that had more than one plot under maize or any of the milpa crops, it was impossible to calculate the yield and had to be discarded from the analysis due to a lack of available plot-level information. The results were further screened for complete crop information and unrealistic values on crop production levels (i.e. ten times higher than the average yield for the different crops—See Supplemental Table 2), resulting in a usable sample of 368 plots.

For statistical analysis, only those crop combinations with a sample size equal to or greater than 9 plots were selected, resulting in 357 plots with, in descending order, the following numbers of 300 plots sown to each cropping combination: maize monocrop (163), maize-bean (109), maize-bean-squash (30), maize-bean/faba (12), maize-potato (13), maize-squash (11), maize-bean-potato (10), maize-faba (9). Other crop combinations with very low sample sizes were maize-squash-faba (4), maize-potato-faba (3), maize-bean-squash-faba (2), maize-bean-potato-faba (1) and maize-bean-potato-squash (1), making it difficult to include them in further statistical analyses.

Maize yields for plots under monocropped maize (163) were first compared to maize yields from intercropped plots (194). To choose the most appropriate statistical test, we checked if the outcome variable, maize yield, met the assumptions required for a parametric test. Although we had independent samples, large sample sizes, and homogeneous variances (F = 1.1809, p-value = 0.267), maize yield proved to be non-normally distributed after Shapiro-Wilks normality test was significant (W = 0.911, p-value < 0.000 (1.398e−13)). Thus, we choose a non-parametric test, the Wilcoxon rank-sum (also known as the independent 2-group Mann–Whitney U Test), to compare maize yields in monocrop and intercrop. Results are shown in boxplots for yield of both groups in Fig. 3-A. Maize yield was also compared between all eight crop associations, as maize yield presented a non-normal distribution and we have several groups to compare, a Kruskal–Wallis test, was performed. While in general, omnibus test like Kruskal–Wallis are used to detect the existence of at least one significant difference across groups, sometimes they fail in detecting significant differences between pairs of groups, and hence we decided to perform a post hoc test. The Dunn’s test of multiple comparisons was selected as the most appropriate one as it is suited for groups with unequal number of observations^27^ and because it retains the rank sums from the omnibus test, this case Kruskal–Wallis^28^. Results of maize yields for the different cropping systems and Dunn’s test significant groups are presented with boxplots in Fig. 4.

### Potential adequacy of milpas for human nutrition

We applied a Functional Diversity approach used in ecology research^29–31^ to assess the nutritional performance of milpa systems. We calculated the number of people who can obtain recommended daily allowances (RDA) of 14 different nutrients from different milpa systems. We then calculated the Potential Nutrient Adequacy (PNA)^18^ to determine the number of persons (male adult equivalents) who could obtain RDAs of a full set of nutrients from 1 ha of a milpa system, including monocropped maize and associations, using the following equation:

$${\text{PNA}} = \frac{{\mathop \sum \nolimits_{{{\text{i}} = 1}}^{{\text{N}}} [{\text{s}}_{{\text{i}}} > 1]{ }}}{{\text{N}}}{ } \times {\overline{\text{s}}}\,{[18]}$$

where $N$ is the number of nutrients considered (14), $s_{i}$ is the fraction of potentially nourished male adults by a given nutrient, and $\overline{s}$ is the average of potentially nourished persons for all nutrients. To calculate *s* we multiplied the yield of each crop reported in the survey (kg ha^−1^) by the nutrient composition of each crop, using the content per 100 g of the 14 different nutrients for each crop, as per the INCAP Food Composition Table for Central America^32^ and using the INCAP RDA for an adult male^33^ (See Supplementary Tables 3 and 4). All values were calculated per hectare and year. PNA levels across cropping associations were also compared. First, we checked if PNA data met the assumptions required for a parametric test. PNA variances proved to be non-homogeneous (F = 14.5, p-value =  < 2e-16) and also have a non-normal distribution (Shapiro–Wilk , W = 0.93742, p-value = 4.107e-11). Thus, a Kruskall-Wallis and Dunn’s tests were performed to assess PNA differences between cropping combinations. Results are presented with boxplots in Fig. 6.

## Supplementary Information

Supplementary Information
