# Supplementary material for: Maize intercropping in the milpa system. Diversity, extent and importance for nutritional security in the Western Highlands of Guatemala
Source: Sci Rep. 2021 Feb 12;11:3696. doi: 10.1038/s41598-021-82784-2 (PMC7881131; doi:10.1038/s41598-021-82784-2)
Supplement: Supplementary file 1 — Supplementary Information [file 41598_2021_82784_MOESM1_ESM.docx]

Supplementary Information

**Diversity and extent of maize intercropping in the milpa system in the Western Highlands of Guatemala and its importance for nutritional security**

Lopez-Ridaura Santiago, Luis Barba-Escoto, Cristian Reyna, Carlos Sum, Natalia Palacios-Rojas and Bruno Gerard

Supplementary Table 1. The 64 Villages surveyed by Buena Milpa Project by Department and Municipality

| Department | Municipality | Village | Freq |
| --- | --- | --- | --- |
| Huehuetenango | Chiantla | El Pino | 20 |
| Huehuetenango | Chiantla | Las Majadas | 14 |
| Huehuetenango | Chiantla | Quilinco | 15 |
| Huehuetenango | Chiantla | San Franciscolas flores | 20 |
| Huehuetenango | Chiantla | San Jose las flores | 20 |
| Huehuetenango | Concepción Huista | Ap | 12 |
| Huehuetenango | Concepción Huista | Cabic | 21 |
| Huehuetenango | Concepción Huista | Canalaj | 20 |
| Huehuetenango | Concepción Huista | Sechec | 19 |
| Huehuetenango | Concepción Huista | Tierra Comun | 20 |
| Huehuetenango | Todos Santos | Caserio Tican | 15 |
| Huehuetenango | Todos Santos | Las lajas | 10 |
| Huehuetenango | Todos Santos | Villa Alicia | 20 |
| Quetzaltenango | Concepción Chiquirichapa | El Aguacate | 20 |
| Quetzaltenango | Concepción Chiquirichapa | Excomuchaj | 6 |
| Quetzaltenango | Concepción Chiquirichapa | Pueblo Nuevo | 1 |
| Quetzaltenango | Concepción Chiquirichapa | San Cristobal | 14 |
| Quetzaltenango | Concepción Chiquirichapa | Tojchan | 16 |
| Quetzaltenango | Concepción Chiquirichapa | Tuipox | 20 |
| Quetzaltenango | Concepción Chiquirichapa | Tuitzisbil | 17 |
| Quetzaltenango | San Juan Ostuncalco | Los Alonzo | 19 |
| Quetzaltenango | San Juan Ostuncalco | Los Romero | 20 |
| Quetzaltenango | San Juan Ostuncalco | Pueblo Nuevo | 14 |
| Quetzaltenango | San Juan Ostuncalco | San Francisco | 20 |
| Quetzaltenango | San Juan Ostuncalco | Varsovia | 20 |
| Quiche | Chajul | Canton Chajul | 17 |
| Quiche | Chajul | Ilom | 18 |
| Quiche | Chichicastenango | Chicua I | 20 |
| Quiche | Chichicastenango | Chucalibal 1 | 25 |
| Quiche | Chichicastenango | Chucalibal ll | 17 |
| Quiche | Chichicastenango | Chuguexa 1 | 17 |
| Quiche | Chichicastenango | Chuitzorop I | 5 |
| Quiche | Chichicastenango | Chupol | 20 |
| Quiche | Chichicastenango | Los Alonzo | 1 |
| Quiche | Chichicastenango | Semeaj | 18 |
| Quiche | Chichicastenango | Xecalibal | 16 |
| Quiche | Chichicastenango | Xepol | 20 |
| Quiche | Chichicastenango | Xetzac | 1 |
| Quiche | Cunen | Centro Cunen | 2 |
| Quiche | Cunen | La Barranca | 14 |
| Quiche | Cunen | Las Vegas | 15 |
| Quiche | Cunen | Los Trigales | 19 |
| Quiche | Cunen | Ojo de Agua | 20 |
| Quiche | Cunen | Santa Clara | 15 |
| Quiche | Cunen | Xetzac | 18 |
| Quiche | Nebaj | El paraiso | 12 |
| Quiche | Nebaj | Kanaquil | 7 |
| Quiche | Nebaj | Palob Chiquito | 13 |
| Quiche | San Juan Cotzal | Chichel | 20 |
| Totonicapan | Momostenango | Chicakuleu | 10 |
| Totonicapan | Momostenango | Chorracana | 19 |
| Totonicapan | Momostenango | Chucabja | 16 |
| Totonicapan | Momostenango | Chukaculeu | 11 |
| Totonicapan | Momostenango | Chumuxol | 15 |
| Totonicapan | Momostenango | Pueblo Viejo | 15 |
| Totonicapan | Momostenango | San Jose Siguila | 18 |
| Totonicapan | Momostenango | Tierra Blanca | 15 |
| Totonicapan | Momostenango | Tierra Colorada | 21 |
| Totonicapan | San Bartolo | Tierra Blanca | 4 |
| Totonicapan | Santa Lucía La Reforma | Chisiguan | 23 |
| Totonicapan | Santa Lucía La Reforma | Oxlajuj | 24 |
| Totonicapan | Santa Lucía La Reforma | Xoltacaj | 17 |
| Totonicapan | Santa Maria Chiquimula | Chicastro | 17 |
| Totonicapan | Santa Maria Chiquimula | Tierra Blanca | 1 |
|  |  | **Total** | 989 |


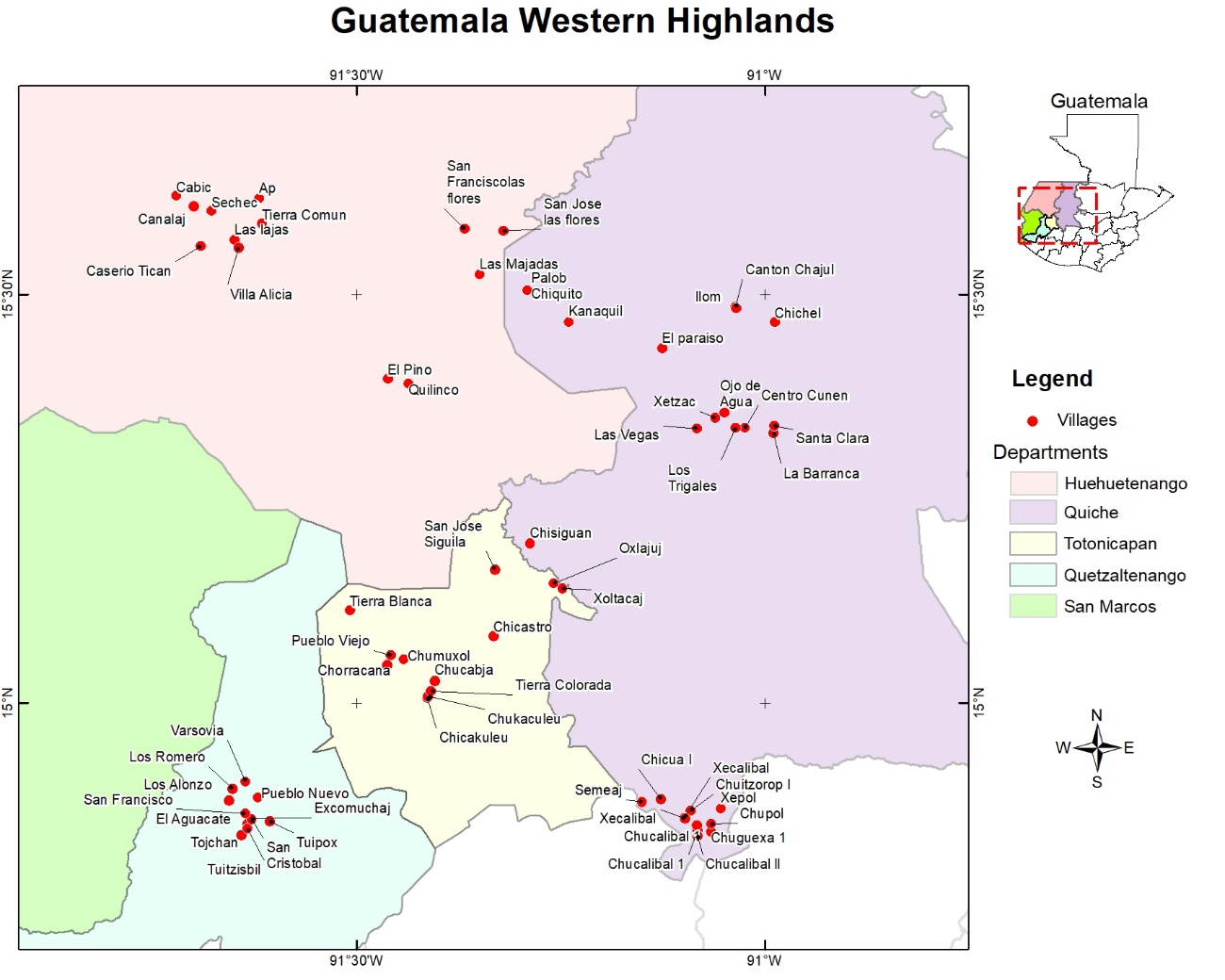


Supplementary Figure 1. Villages in the Western Highlands of Guatemala that were covered in a survey of 989 maize-growing households, 2015 (Created with Esri ArcMap TM 10.4.1.5686 from ArcGis 10.4.1 for Desktop https://www.esri.com/en-us/home).

Supplementary Table 2. Crops yield (kg ha^-1^) for diverse maize-based cropping systems in the Western Highlands of Guatemala, 2015.

|  | **Crop Yield in kg ha^-1^ yr^-1^**  **Mean**  **(Std Dev)** | | | | |
| --- | --- | --- | --- | --- | --- |
| Crop Association | **Maize** | **Bean** | **Faba** | **Potato** | **Squash** |
| Maize (N=163) | 1981 (1099) | - | - | - | - |
| MaizeBean (N=109) | 1826 (986) | 256 (337) | - | - | - |
| Maize Bean Faba (N=12) | 2320 (1265) | 140 (108) | 171 (153) | - | - |
| Maize Bean Potato (N=10) | 1755 (800) | 259 (326) | - | 3087 (2054) | - |
| Maize Bean Squash (N=30) | 1651 (1073) | 288 (398) | - | - | 233 (177) |
| Maize Faba (N=9) | 1879 (947) | - | 245 (261) | - | - |
| Maize Potato (N=13) | 1233 (672) | - | - | 4418 (3321) | - |
| Maize Squash (N=11) | 2065 (1175) | - | - | - | 286 (130) |

Supplementary Table 3. Nutrient content of each crop in 100g (Menchu et al. 2012)

|  |  | Crop | | | | |
| --- | --- | --- | --- | --- | --- | --- |
| Nutrient | Unit | Maize | Bean | Squash | Potato | Faba Bean |
| Energy | kcal | 361 | 343 | 30 | 77 | 341 |
| Protein | g | 6.93 | 22.7 | 0.6 | 2.02 | 26.12 |
| Fat | g | 3.86 | 1.6 | 0.2 | 0.09 | 1.53 |
| Carbohydrates | g | 76.85 | 61.6 | 7.6 | 14.47 | 58.3 |
| Dietetic Fiber | g | 13.4 | 18.37 | 0.5 | 2.2 | 25 |
| Vitamin A Retinol | µg | 0 | 0 | 143 | 0 | 3 |
| Vitamin C | mg | 0 | 1 | 15 | 20 | 1 |
| Thiamin | mg | 0.25 | 0.47 | 0.04 | 0.08 | 0.6 |
| Rivoflavin | mg | 0.08 | 0.15 | 0.04 | 0.03 | 0.3 |
| Niacin | mg | 1.9 | 2.09 | 0.5 | 1.05 | 2.8 |
| Folate | µg | 25 | 0 | 16 | 16 | 423 |
| Vitamin B6 | mg | 0.37 | 0.53 | 0.06 | 0.3 | 0.4 |
| Calcium | mg | 7 | 134 | 19 | 12 | 103 |
| Potassium | mg | 315 | 1464 | 340 | 421 | 1062 |
| Iron | mg | 2.38 | 7.1 | 0.5 | 0.8 | 6.7 |
| Zinc | mg | 1.73 | 2.55 | 0.32 | 0.3 | 3.1 |

Supplementary Table 4. Recommended Daily Allowances (RDA) for an adult male between 19-59 years old

| Nutrient | Unit | INCAP values |
| --- | --- | --- |
| Energy | kcal | 2650 |
| Protein | g | 68 |
| Fat | g | 74.2 |
| Carbohydrates | g | 397.5 |
| Dietetic Fiber | g | 21.2 |
| Ash | - | - |
| Calcium | mg | 1000 |
| Phosphorus | mg | 800 |
| Iron | mg | 11 |
| Thyamin | mg | 1.2 |
| Rivoflavin | mg | 1.5 |
| Niacin | mg | 20 |
| Vitamin C | mg | 60 |
| Vitamin A | ug | 600 |
| Fatty Acids Mono Unsaturated | - | - |
| Fatty Acids Poly Unsaturated | - | - |
| Fatty Acids Saturated | - | - |
| Potassium | mg | 2000 |
| Sodium | mg | 575 |
| Zinc | mg | 18 |
| Magnesium | mg | 310 |
| Vitamin B6 | mg | 1.4 |
| Folic Acid | - | - |
| Folate | ug | 200 |
